# Supplementary material for: BNT162b2 vaccination enhances interferon-JAK-STAT-regulated antiviral programs in COVID-19 patients infected with the SARS-CoV-2 Beta variant
Source: Commun Med (Lond). 2022 Feb 22;2:17. doi: 10.1038/s43856-022-00083-x (PMC9029844; doi:10.1038/s43856-022-00083-x)
Supplement: Supplementary file 1 — Description of Additional Supplementary Files [file 43856_2022_83_MOESM1_ESM.pdf]

Supplementary Data

Supplementary Data 1. Demographic and Clinical Characteristics of study population.

Supplementary Data 2. List of all genes with normalized read counts in each replicate at unvaccinated and vaccinated COVID-19 patients who were infected by Beta variant, $\log_2$ (fold change), *p*-value and adjusted *p*-value as well as upregulated gene list and GSEA analysis.

Supplementary Data 3. List of all genes with normalized read counts in each replicate at longitudinal study of vaccinated group, $\log_2$ (fold change), *p*-value and adjusted *p*-value as well as upregulated gene list and GSEA analysis.

Supplementary Data 4. List of all genes with normalized read counts in each replicate at longitudinal study of unvaccinated group, $\log_2$ (fold change), *p*-value and adjusted *p*-value as well as upregulated gene list and GSEA analysis.

Supplementary Data 5. List of all genes with normalized read counts in each replicate at Day0 (prior to vaccination) and Day7 or 10 from the BNT162b vaccination of naïve individuals, $\log_2$ (fold change), *p*-value and adjusted *p*-value as well as upregulated gene list and GSEA analysis.

Supplementary Data 6. List of known OAS1 SNPs that were detected in bam file of RNA-seq for each patient.

Supplementary Data 7. List of all genes with normalized read counts in each replicate at Day0 (prior to vaccination) and the first samples of the unvaccinated patients, $\log_2$ (fold change), *p*-value and adjusted *p*-value as well as upregulated gene list and GSEA analysis.

Supplementary Data 8. List of all genes with normalized read counts in each replicate at Day0 (prior to vaccination) and the first samples of the vaccinated patients, $\log_2$ (fold

change), *p*-value and adjusted *p*-value as well as upregulated gene list and GSEA analysis.
