# Supplementary material for: BNT162b2 vaccination enhances interferon-JAK-STAT-regulated antiviral programs in COVID-19 patients infected with the SARS-CoV-2 Beta variant
Source: Commun Med (Lond). 2022 Feb 22;2:17. doi: 10.1038/s43856-022-00083-x (PMC9029844; doi:10.1038/s43856-022-00083-x)
Supplement: Supplementary file 2 — Supplementary information [file 43856_2022_83_MOESM2_ESM.pdf]

Supplementary Information

BNT162b2 vaccination enhances interferon-JAK-STAT-regulated antiviral programs in COVID-19 patients infected with the SARS-CoV-2 Beta variant

Ludwig Knabl[1,*,†], Hye Kyung Lee[2,*,†], Manuel Wieser[1], Anna Mur[3], August Zabernigg[3], Ludwig Knabl Sr.[4], Simon Rauch[5], Matthias Bock[5, 6], Jana Schumacher[7], Norbert Kaiser[7], Priscilla A. Furth[8,†] and Lothar Hennighausen[2,†]

[1]TyrolPath, Zams, Austria; [2]National Institute of Diabetes, Digestive and Kidney Diseases, Bethesda, MD 20892, USA; [3]Division of Internal Medicine, Krankenhaus Kufstein, Kufstein, Austria; [4]Krankenhaus St. Vinzenz, Zams, Austria; [5]Division of Anesthesia and Intensive Care Medicine, Krankenhaus Meran, Meran, Italy; [6]Department of Anesthesiology, perioperative Medicine and Intensive Care Medicine, Paracelsus Medical University, Salzburg, Austria; [7]Division of Internal Medicine, Krankenhaus St. Johann, St. Johann, Austria; [8]Departments of Oncology & Medicine, Georgetown University, Washington, DC, USA.

*These authors contributed equally.

[†]Corresponding authors: LK: Ludwig.knabl@tyrolpath.at; HKL: hyekyung.lee@nih.gov; PAF: paf3@georgetown.edu; LH: lotharh@nih.gov

Supplementary Figures

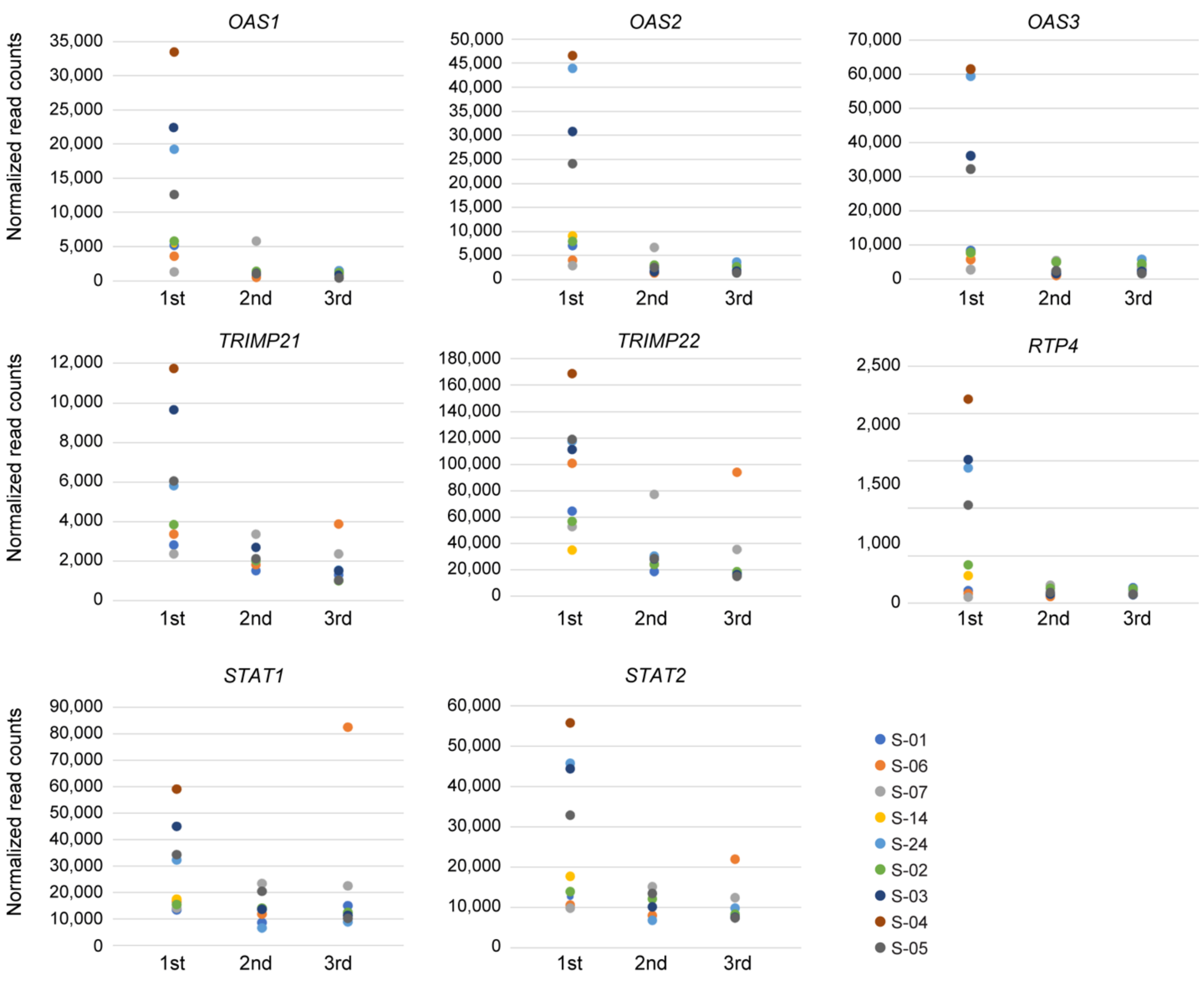

Supplementary Fig. 1. Upregulation of OASs and ISGs genes in COVID-19 patients. Dots show the normalized read counts of each patient. mRNA levels of antiviral and JAK/STAT signaling components, OAS1/2/3, Trimp21/22, RTP4 and STAT1/2 were measured by RNA-seq.

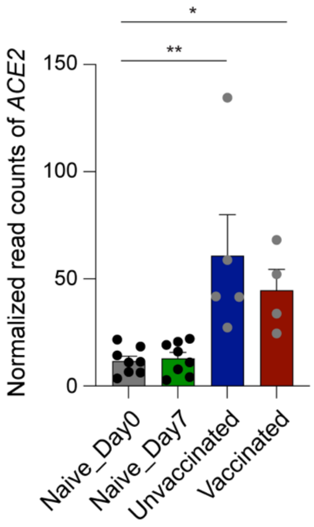

Supplementary Fig. 2. Relative normalized expression of ACE2 gene from 1st samples of unvaccinated and vaccinated patients as well as Day 0 and Day 7-10 of naïve volunteers receiving the first dose of the BNT162b2 vaccine. A one-way ANOVA with Dunnett's multiple comparisons was used to evaluate the statistical significance between two groups as well as two time points. *$p < 0.05$, **$p < 0.001$.

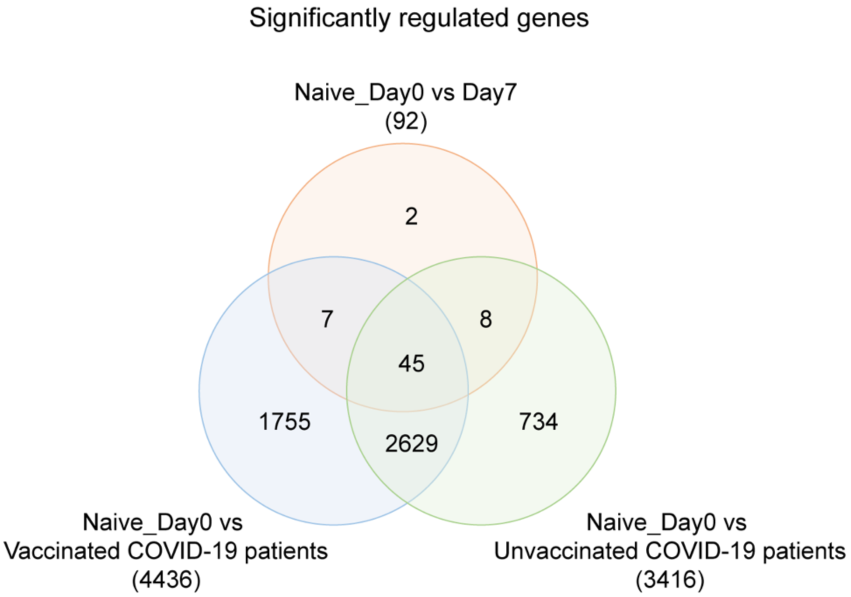

Supplementary Fig. 3. Venn diagrams showing overlap of significantly regulated genes between comparisons of Naïve_Day0 vs Naïve_Day7, Naïve_Day0 vs Vaccinated COVID-19 patients, and Naïve_Day0 vs Unvaccinated COVID-19 patients.

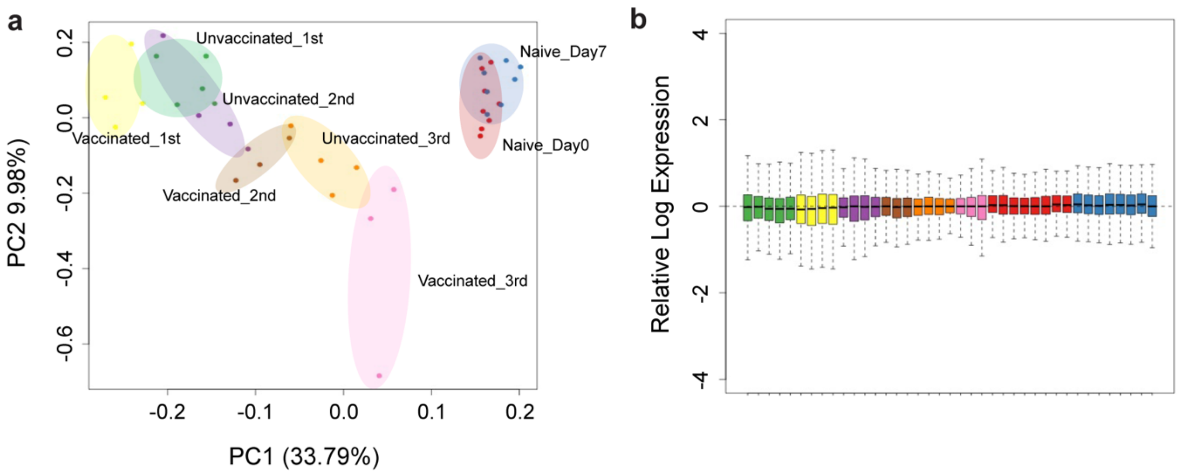

Supplementary Fig. 4. PCA (a) and RLE (b) analysis of bulk RNA-seq samples.
