# Supplementary material for: BNT162b2 vaccination enhances interferon-JAK-STAT-regulated antiviral programs in COVID-19 patients infected with the SARS-CoV-2 Beta variant
Source: Commun Med (Lond). 2022 Feb 22;2:17. doi: 10.1038/s43856-022-00083-x (PMC9029844; doi:10.1038/s43856-022-00083-x)
Supplement: Supplementary file 12 — Reporting Summary [file 43856_2022_83_MOESM12_ESM.pdf]

nature research

Corresponding author(s): Lothar Hennighausen

Last updated by author(s): Jan 26, 2022

Reporting Summary

Nature Research wishes to improve the reproducibility of the work that we publish. This form provides structure for consistency and transparency in reporting. For further information on Nature Research policies, see our Editorial Policies and the Editorial Policy Checklist.

Statistics

For all statistical analyses, confirm that the following items are present in the figure legend, table legend, main text, or Methods section.

n/a	Confirmed	
☐	☒	The exact sample size (n) for each experimental group/condition, given as a discrete number and unit of measurement
☐	☒	A statement on whether measurements were taken from distinct samples or whether the same sample was measured repeatedly
☐	☒	The statistical test(s) used AND whether they are one- or two-sided<br>*Only common tests should be described solely by name; describe more complex techniques in the Methods section.*
☒	☐	A description of all covariates tested
☐	☒	A description of any assumptions or corrections, such as tests of normality and adjustment for multiple comparisons
☐	☒	A full description of the statistical parameters including central tendency (e.g. means) or other basic estimates (e.g. regression coefficient) AND variation (e.g. standard deviation) or associated estimates of uncertainty (e.g. confidence intervals)
☐	☒	For null hypothesis testing, the test statistic (e.g. F, t, r) with confidence intervals, effect sizes, degrees of freedom and P value noted<br>*Give P values as exact values whenever suitable.*
☒	☐	For Bayesian analysis, information on the choice of priors and Markov chain Monte Carlo settings
☒	☐	For hierarchical and complex designs, identification of the appropriate level for tests and full reporting of outcomes
☒	☐	Estimates of effect sizes (e.g. Cohen's d, Pearson's r), indicating how they were calculated

Our web collection on statistics for biologists contains articles on many of the points above.

Software and code

Policy information about availability of computer code

Data collection	No software was used for data collection.
Data analysis	The raw data were subjected to QC analyses using the FastQC tool (version 0.11.9). mRNA-seq read quality control was done using Trimmomatic (version 0.36) and STAR RNA-seq (version STAR 2.5.4a) using 150 bp paired-end mode was used to align the reads (hg19). HTSeq (version 0.9.1) was to retrieve the raw counts. R and DESeq2 were used for differential gene analysis and the Wald statistic. Additionally, the RUVSeq package was applied to remove unwanted variation (RUV). The data were pre-filtered keeping only genes with at least ten reads in total. The visualization was done using dplyr and ggplot2.

For manuscripts utilizing custom algorithms or software that are central to the research but not yet described in published literature, software must be made available to editors and reviewers. We strongly encourage code deposition in a community repository (e.g. GitHub). See the Nature Research guidelines for submitting code & software for further information.

Data

Policy information about availability of data

All manuscripts must include a data availability statement. This statement should provide the following information, where applicable:

- Accession codes, unique identifiers, or web links for publicly available datasets
- A list of figures that have associated raw data
- A description of any restrictions on data availability

ChIP-seq and RNA-seq data from primary bronchial cells were obtained from GSE161665. In addition, we analyzed the ChIP-seq for STATs from GSE31477. The RNA-seq data of COVID-19 patients and vaccinated volunteers investigated in this study were uploaded under the accession GSE189039.

Field-specific reporting

Please select the one below that is the best fit for your research. If you are not sure, read the appropriate sections before making your selection.

☒ Life sciences ☐ Behavioural & social sciences ☐ Ecological, evolutionary & environmental sciences

For a reference copy of the document with all sections, see nature.com/documents/nr-reporting-summary-flat.pdf

Life sciences study design

All studies must disclose on these points even when the disclosure is negative.

Sample size	Sample size was determined based on availability and resources. However, we analyzed power analysis for sample size used in this study and it was summarized in the Method. 9 COVID-19 patients and 8 vaccinated individuals were enrolled based on recruitment, and they provided blood samples.
Data exclusions	No data were excluded.
Replication	Human samples were collected from 17 participants at several time points. There were no duplicate from a certain time point.
Randomization	Randomization was not relevant to this study as it represents a fully observational study.
Blinding	Blinding was not relevant to this study as patients were previously categorized on the result of SARS-CoV-2 test.

Reporting for specific materials, systems and methods

We require information from authors about some types of materials, experimental systems and methods used in many studies. Here, indicate whether each material, system or method listed is relevant to your study. If you are not sure if a list item applies to your research, read the appropriate section before selecting a response.

Materials & experimental systems

n/a	Involved in the study	
☒	☐	Antibodies
☒	☐	Eukaryotic cell lines
☒	☐	Palaeontology and archaeology
☒	☐	Animals and other organisms
☐	☒	Human research participants
☒	☐	Clinical data
☒	☐	Dual use research of concern

Methods

n/a	Involved in the study	
☒	☐	ChIP-seq
☒	☐	Flow cytometry
☒	☐	MRI-based neuroimaging

Human research participants

Policy information about studies involving human research participants

Population characteristics	Study participants demographics are detailed in Supplementary Data 1.
Recruitment	Study participants were recruited from Tyrol, Austria by multiple hospitals that are listed in affiliation. Potential self-selection and recruiting biases are unlikely to affect the parameters we measured.
Ethics oversight	This study was approved by the Institutional Review Board (IRB) of the Office of Research Oversight / Regulatory Affairs, Medical University of Innsbruck, Austria (EC numbers: 1064/2021). A written informed consent was obtained from all study participants. The informed consent was checked and approved by the IRB of the Office of Research Oversight/Regulatory Affairs, Medical University of Innsbruck, Austria.

Note that full information on the approval of the study protocol must also be provided in the manuscript.
